# Cross-cultural adaptation and validation of the Spanish version of the American Academy of Orthopaedic Surgeons-Foot and Ankle Module (AAOS-FAMsp)

**DOI:** 10.1186/s13018-016-0409-7

**Published:** 2016-07-06

**Authors:** Manuel González-Sánchez, Esther Velasco-Ramos, Maria Ruiz-Muñoz, Antonio I. Cuesta-Vargas

**Affiliations:** Health Science Faculty, Department of Health Science, University of Jaén, Campus de las Lagunillas SN. Ed.B3 - Despacho 066, 23071 Jaén, Spain; Health Science Faculty, Universidad de Málaga, Málaga, Spain; Departamento de Enfermería y Podología, Instituto de Investigación Biomédica de Málaga (IBIMA), Universidad de Málaga, Málaga, Spain; Departamento de Psiquiatria y Fisioterapia, Instituto de Investigación Biomédica de Málaga (IBIMA), Universidad de Málaga, Málaga, Spain; School of Clinical Sciences at Queensland University, Brisbane, Australia

**Keywords:** Foot, Patient reported outcome measures, Assessment, Spanish version, Validation

## Abstract

**Background:**

The current study performed a cross-cultural adaptation to Spanish and examined the internal and external validation of the AAOS-FAM questionnaire.

**Methods:**

A direct translation (English to Spanish) and a reverse translation (Spanish to English) were performed by two independent professional native translators. Cronbach’s α coefficients were calculated to analyse the internal consistency of the measure. The factor structure and construct validity were analysed after extraction by maximum likelihood (EML); extraction was necessary if the following three requirements were met: accounting for ≥10 % of variance, Eigenvalue >1.0 and a scree plot inflexion point. The standard error of measurement and minimal detectable change 90 (MDC90) were calculated. Criterion validity was calculated by analysing the correlation between the American Academy of Orthopaedic Surgeons-Foot and Ankle Module (Spanish version) (AAOS-FAMsp) and Spanish versions of the questionnaires FFI and FHSQ.

**Results:**

Regarding internal consistency, Cronbach’s α was 0.877, and in the test-retest analysis, the ICC ranged between 0.899 and 0.942. Error measures were calculated by MDC90 and SEM, which showed values of 3.444 and 1.476 %, respectively. The analysis demonstrated a goodness of fit chi-squared value of 803.166 (*p* < 0.001). For criterion validity, the correlation value with FFIsp was *r* = 0.837 (*p* < 0.01), while the FHSQsp correlation values with different scales ranged from *r* = 0.206 (*p* < 0.01) (physical activity) to *r* = 0.665 (*p* < 0.01) (pain).

**Conclusions:**

The results indicate that AAOS-FAMsp has satisfactory psychometric properties, facilitating the inclusion of Spanish-speaking individuals into both research and clinical practice.

**Electronic supplementary material:**

The online version of this article (doi:10.1186/s13018-016-0409-7) contains supplementary material, which is available to authorized users.

## Background

In the last 20 years, patient reported outcome measures (PROM) have emerged as an important way to assess and monitor patients and currently are widely used in clinical practice and research [1, 2]. These instruments are inexpensive, easy to use and specific and reliable tools. They facilitate the determination of a patient’s health and functional status and the interpretation of results for clinicians, researchers and patients regarding a patient’s symptoms, capabilities and/or functioning [1, 3, 4].

Given the structure and function of the foot, any problematic condition in the foot may have a profound negative impact on a patient’s quality of life and function [5]. With the intention of assessing the impact of foot problems in a patient, the American Academy of Orthopaedic Surgeons developed a specific module for the subjective assessment of changes in the foot, i.e. the Foot and Ankle Module (AAOS-FAM) [6]. This questionnaire has two scales, the Global Foot and Ankle Scale and the Foot Comfort Scale, comprised of 25 items in total, with a retest reliability between 0.79 and 0.99 [6]. Until now, the AAOS-FAM questionnaire has not been a translation or culturally adapted into Spanish, one of the five most widely spoken languages in the world [7, 8] and an official language of the United Nations [9]. A cultural adaptation and validation of the AAOS-FAM Spanish questionnaire was conducted in this study to facilitate the collection of clinical data from Spanish-speaking individuals and to help improve the standardisation of data collection in clinical research and treatment throughout the country.

The aim of this study was to perform a cross-cultural adaptation to Spanish and to examine the internal and external validation of the AAOS-FAM questionnaire, with the intention of facilitating the inclusion of Spanish-speaking individuals into both research and clinical practice.

## Methods

### Design

This observational study was conducted with patients recruited from three public and private podiatric clinics in southern Spain. One hundred and ninety-three (193) patients (99 women and 94 men) with a mean age of 55.49 (±16.10) years participated in the present study (Table 1). The inclusion criteria for the participants were as follows: native Spanish speaker, aged 18 years old or older, having an altered foot that requires treatment and able to walk independently. Participants with a cognitive impairment of any aetiology that prevented them from completing the questionnaires independently were excluded from the present study.

**Table 1:** Descriptive anthropometric data of the sample

		Mean	SD	Minimum	Maximum
Age (years)		55.49	16.10	21	88
Weight (kg)		61.22	9.37	48.22	94.34
Height (cm)		167.70	9.53	151.30	187.20
BMI (kg/m^2^)		22.95	2.19	19.68	31.44
Hours weekly’s standing		45.76	6.91	20	65
N		193			
Gender (female/male)		99/94			
Education level	Elementary	49			
	Secondary	86			
	University	47			
	Master/PhD	11			
Laterality (right/left)		158/35			

### Translation and transcultural adaptation of AAOS-FAM to AAOS-FAMsp

**Fig. 1:**
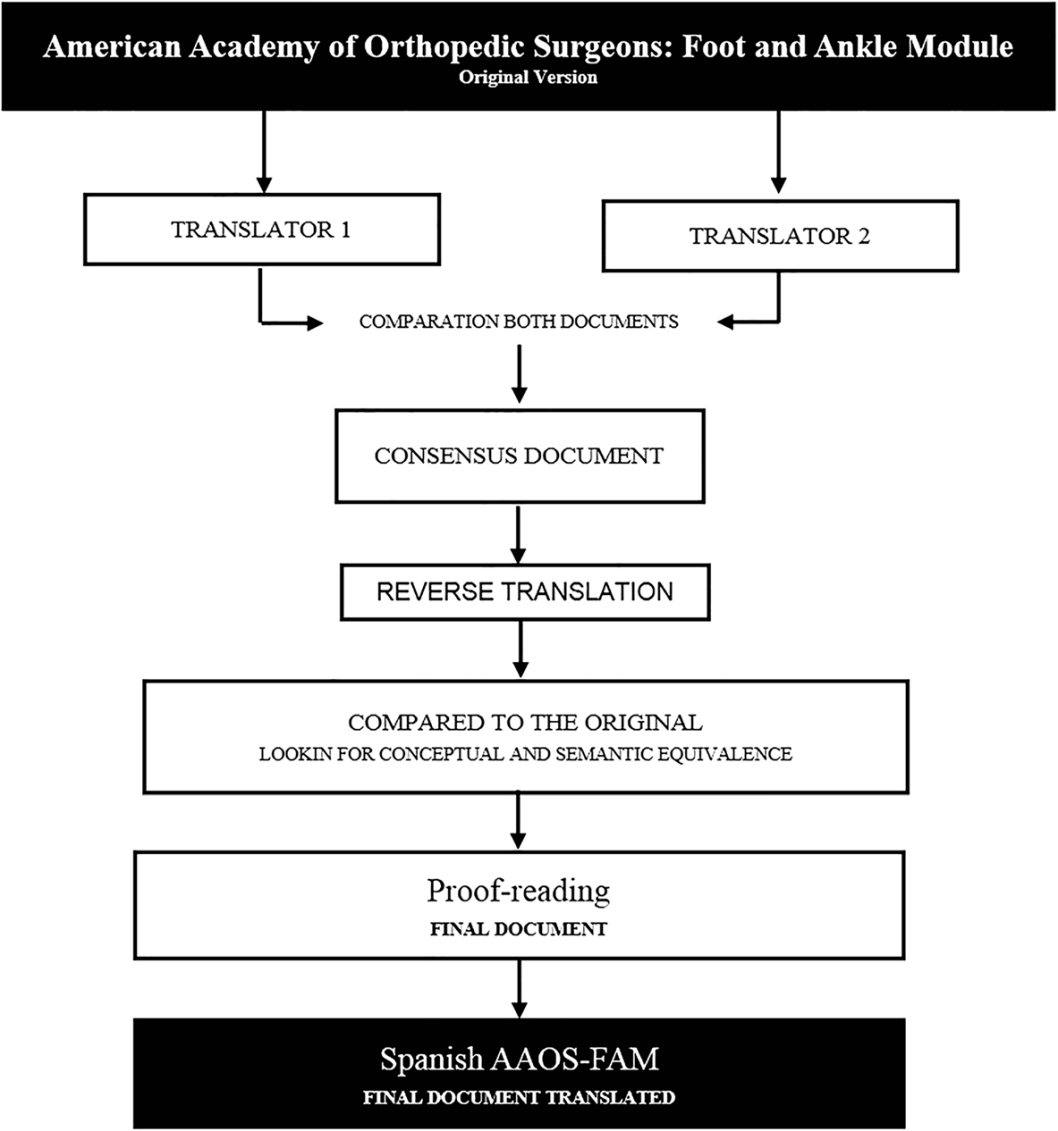
Flowchart of the development of AAOS-FAMsp from the original version

The process of translating the original AAOS-FAM to the American Academy of Orthopaedic Surgeons-Foot and Ankle Module (Spanish version) (AAOS-FAMsp) questionnaire was carried out in different phases which are summarised in Fig. 1. Direct translation (English to Spanish) and the reverse translation (Spanish to English) were performed by two independent professional native translators. With the aim of ensuring the conceptual equivalence of the terms used, a translation process was performed, as recommended by the literature [10–12].

### Data collection

Between 1 February 2015 and 31 May 2015, all subjects included as participants completed the following questionnaires: AAOS-FAMsp, Foot Function Index (FFI) and Foot Health Status Questionnaire (FHSQ). In order to calculate the reliability of the AAOS-FAMsp, this questionnaire was completed a second time 4 days later. This period of time was used to ensure the condition of the participants had not changed between measurements [13].

The AAOS-FAM consists of 25 questions comprising two scales: the Global Foot and Ankle Scale and the Foot Comfort Scale. The first of the 20-item scales is used to test foot function, inflammation, pain and stiffness, which generated a single score between 0 and 100. The second scale, consisting of five questions, is used to assess comfort in terms of wearing shoes (with a yes or no for each type), which generated a scale ranging from 0 to 100 (0 = poor outcome and 100 = the best possible outcome) [6]. The two scales are combined to provide an index ranging from 0 to 100. We weighed each scale in the final score based on the number of items: Global Foot and Ankle Scale 80 % (20 items) and Foot Comfort Scale 20 % (five items) [6].

The original version of the FFI questionnaire consists of 23 questions [14, 15]. Each question is answered on a visual analogue scale, ranging from 0 to 9. If patients cannot respond to any question, they are instructed to leave it blank and it is not included in the final score of the questionnaire [14–16]. The final result is offered on a scale from 0 to 100, in which all the questions are summed and then divided by 207 (the highest possible score, i.e. 23 × 9) and multiplied by 100 and then rounded up, if necessary, to give an integer between 0 and 100 [16]. The cross-cultural adaptation of the Spanish FFI questionnaire was validated and published by Paez-Moguer et al. (2013) [16].

The FHSQ is an instrument designed to measure the quality of life related to the health of the feet [17–19]. The 19 questions evaluate four domains of foot health: pain, function, general health and footwear. Each is rated on a Likert (numerical) style 0–100 scale, where 0 is the worst health status and 100 indicates the best health status. This was validated by Bennett and Patterson to evaluate the effectiveness of surgical and conservative treatment of diseases involving the skin and nails, as well as neurological, musculoskeletal and orthopaedic disorders [17–19]. Spanish cross-cultural adaptation of the FHSQ questionnaire was validated and published by Cuesta-Vargas et al. (2013) [17].

### Data analysis

A descriptive analysis of the anthropometric variables and the characteristics of participants was conducted. The Kolmogorov-Smirnov test was used to analyse the distribution and normality of the sample. Cronbach’s α coefficients were calculated to analyse the internal consistency of measures by classifying the values according to the following scale: Cronbach’s α ≤0.40 (poor), 0.60 > Cronbach’s α > 0.40 (moderate), 0.80 > Cronbach’s α ≥ 0.60 (good) and Cronbach’s α ≥0.80 (excellent) [20]. To analyse whether item performance was similar between men and women, a comparison of the variables between genders was conducted. All variables presented a parametric distribution, and for this reason, Student’s *t* test was used to calculate the differences between groups.

The factor structure and construct validity were analysed after extraction by maximum likelihood (EML); extraction was necessary if the following three requirements were met: accounting for >10 % of variance, Eigenvalue >1.0 and a scree plot inflexion point.

The formula $\mathrm{S}\mathrm{E}\mathrm{M}=\mathrm{s}\sqrt{1-r}$ was used to calculate the standard error of measurement (SEM), where *s* is the standard deviation (SD) of the test score for both measurements (time 1 and 2) and *r* is the reliability coefficient for the test and interclass correlation (ICC) between test and retest values.

Following the analysis described by Stratford [21], the minimal detectable change 90 (MDC90) was used to measure the sensitivity or measurement error. The formula used for the calculation was:

$$ \mathrm{M}\mathrm{D}\mathrm{C}90=\mathrm{S}\mathrm{E}\mathrm{M}\times \sqrt{2 \times 1.65}. $$

Criterion validity was calculated by analysing the correlation between the AAOS-FAMsp and Spanish versions of the questionnaires FFI [16] and FHSQ [17]. The Pearson correlation coefficient was interpreted according to the following scale: *r* ≤ 0.49 (poor), 0.50 ≤ *r* ≤ 0.74 (moderate) and *r* ≥ 0.75 (strong) [22].

The minimum power required to develop strength-related criterion validity of the AAOS-FAMsp indicated a minimum number of 108 subjects, calculated for a 15 % attrition rate with *p* < 0.05 [12]. The statistical analyses were performed using the statistical analysis programme SPSS (v.17.0).

## Results

The AAOS-FAM was translated into Spanish and culturally adapted to provide the new AAOS-FAMsp (available in Additional file 1). Table 1 shows the descriptive data of the sample, which included anthropometric data and the number of hours the participant stands during the week. The average value of the AAOS-FAMsp was 45.66 (±7.38); the mean values of the respective scales were 46.02 (±8.43) (Global Foot and Ankle Scale) and 44.20 (±8.25) (Foot Comfort Scale). No significant gender differences emerged when comparing the responses per item. For internal consistency, Cronbach’s α was 0.877, and in the test-retest analysis, the ICC ranged between 0.899 and 0.942. Error measures were calculated by MDC90 and SEM, with values of 3.444 and 1.476 %, respectively.

**Fig. 2:**
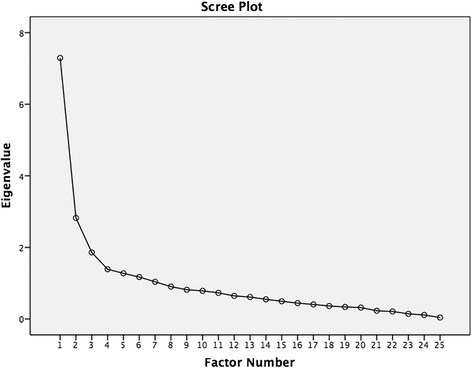
Scree plot of the exploratory factor analysis: two-factor solution

Based on the values observed in Bartlett’s test of sphericity (*p* < 0.001) and the Kaiser-Meyer-Olkin values (0.816), the correlation matrix of the AAOS-FAMsp was deemed adequate for EML. Figure 2 shows the scree plot, where a two-factor solution can be observed. Importantly, there were five factors that had Eigenvalues >1.0, explaining 67.411 % of the total variance; however, they did not explain more than 10 % of the variance, meaning they could not be extracted. In the analysis of the loaded factors, not all the items loaded in the same way for each of the extracted factors (Table 2). Particularly significant were questions 2, 5, 9, 10, 19, 21, 22 and 23, which did not load on either extracted factor, indicating that they can be removed from the questionnaire. The analysis demonstrated a goodness of fit chi-squared value of 803.166 (*p* < 0.001).

**Table 2:** Load distribution of the different items on the factors identified following exploratory factor analysis

		Factor	
		1	2
P7	Did foot ankle give way during strenuous activity	0.947	−0.139
P8	Did foot ankle give way during moderate activity	0.971	−0.131
P13	Pain when doing strenuous activity	0.729	0.039
P14	Pain when doing moderate activity	0.734	0.172
P1	During the past week how stiff was foot ankle	−0.028	0.484
P3	Pain when walking on uneven surfaces	0.388	0.700
P4	Pain when walking on flat surfaces	0.219	0.775
P6	Pain when lying in bed at night	0.292	0.428
P11	How much trouble with balance during the past week	0.263	0.416
P12	How difficult was it to put on or take off socks stockings	0.369	0.437
P15	Pain when doing light activity	0.335	0.635
P16	Pain when standing for an hour	0.366	0.487
P17	Pain when standing for a few minutes	0.215	0.569
P18	How much difficulty walking on uneven surfaces	0.423	0.567
P20	Can wear most shoes	0.136	0.398
P24	How much did ankle foot interfere with normal work	0.211	0.727
P25	How much did ankle foot interfere with life	0.246	0.750
P2	During the past week, how swollen was foot ankle	0.130	0.249
P5	Pain when going up or down stairs	0.020	0.341
P9	Did foot ankle give way during light activity	0.126	0.170
P10	Which statements best describes your ability to get	0.242	0.254
P19	Can wear any shoe	0.207	0.300
P21	Can wear sneakers walking casual shoes	0.064	0.224
P22	Can wear orthopaedic prescription shoes	−0.074	0.020
P23	Can wear all shoes	0.190	0.252

For criterion validity, the correlation value with FFIsp was *r* = 0.837 (*p* < 0.01), while the FHSQsp correlation values with different scales ranged from *r* = 0.206 (*p* < 0.01) (physical activity) to *r* = 0.665 (*p* < 0.01) (pain) (Table 3). Moreover, the higher correlation occurred between each of the subscales as well as with the final score of AAOS-FAMsp.

**Table 3:** Correlation matrix between AAFOS-FAMsp and subscales as well as FHSQ subscales and FFI

		AAOS-FAMsp	Global foot and ankle scale	Shoe comfort scale
AAOS-FAMsp	AAOS FAMsp	1		
	Global Foot and Ankle Scale	0.977***	1	
	PRE Shoe Comfort Scale	0.477**	0.277*	1
FHSQ subscales	FHSQ shoe	0.545*	0.428*	0.690***
	FHSQ foot function	0.622***	0.605*	0.310***
	FHSQ foot pain	0.665***	0.653*	0.304*
	FHSQ GFH	0.596***	0.598*	0.219*
	FHSQ general health	0.426**	0.434**	0.131
	FHSQ physical activity	0.206*	0.216*	0.035
	FHSQ social capacity	0.409**	0.402**	0.187*
	FQSQ vigour	0.298*	0.337**	−0.048
FFI		0.837***	0.799***	0.474*

Significance: *≤0.05; **≤0.005; ***≤0.001

## Discussion

The process of translating and culturally adapting the AAOS-FAMsp ensures the conceptual equivalence of terms used between the original questionnaire and the final version of the AAOS-FAMsp, facilitating its introduction and use among native speakers of the second most widely spoken language in the world. In addition, an analysis of the psychometric properties of the questionnaire, including the criterion validity, construct validity, internal consistency and reliability of the measurement was performed, and the authors found optimal psychometric properties as well as high internal consistency and reliability with a strong correlation with FFIsp. These results indicate that it can be used for assessment and monitoring among Spanish speakers to facilitate obtaining clinical results of high quality for the evaluation of the foot-ankle joint. Therefore, the aim of this study was achieved.

The process of adapting the Spanish AAOS-FAM was conducted following suggestions in the literature [11, 12] and the procedure developed in previous studies that adapted Spanish-specific questionnaires for different body parts such as the upper limbs [3], back [10], lower limbs [1] or ankle and foot [16, 17], using independent and native translators who assured the equivalence of the terms used in the original questionnaire. Cross-cultural adaptation of the AAOS-FAMsp allows clinicians to use this tool to assess the foot-ankle region.

An exploratory factor analysis was performed with the results of the AAOS-FAMsp. After conducting the exploratory factor analysis, not all items loaded on the factor model, as indicated by the questionnaire score (questions: 1–18, 24, 25 (Global Foot and Ankle Scale) and 19–23 (Foot Comfort Scale)) [6]. In addition, there were some items that clearly did not load on any of the factors that had originally been allocated for the calculation of the two subscales of the AAOS-FAMsp (Global Foot and Ankle Scale and Foot Comfort Scale), as shown in Table 2. However, a confirmatory factor analysis was not performed, as our study did not meet the minimum sample size (10 subjects per item analysed) or the optimal sample size (20 subjects per item analysed) necessary to ensure reliable results [23].

The AAOS-FAMsp demonstrated excellent internal consistency; Cronbach’s α was 0.877, and the test-retest (ICC) per item ranged between 0.899 and 0.942. Although both had excellent Cronbach’s α, the value of AAOS-FAMsp (0.877) was slightly higher than the original version (0.83) [6]. However, the test-retest values were consistent between the two versions (0.899–0.942 for AAOS-FAMsp and 0.92 for the original AAOS-FAM [6]).

The AAOS-FAMsp version used the Spanish versions of the FFI and FHSQ questionnaires for criterion validity, while the original version that performed the analysis used the Lower Limb Core questionnaire and SF-36 Physical Health as references. This was based on the classification made by Field [22]. The correlation with FFIsp was strong (*r* = 0.837), ranging from poor (*r* = 0.206 (physical activity)) to moderate (*r* = 0.665 (pain)) in the FHSQsp subscales (Table 3), whereas the original version presented a moderate correlation (*r* = 0.56) with the SF-36 and a strong correlation (*r* = 0.97) with the Lower Limb Core questionnaire [6].

This study was developed following recommendations in the literature regarding the number of subjects required to conduct a psychometric analysis of the questionnaire, where a minimum of five subjects per item under review is required. The AAOS-FAMsp consists of 25 questions, so a minimum number of 125 subjects would be required; this study included 193 participants [24]. However, this study had some limitations, as not all items loaded onto the two factors described within the original questionnaire (Global Foot and Ankle Scale and Shoe Comfort Scale), so future studies could develop a modified version of the AAOS-FAMsp according to the factors identified in this study. Moreover, no psychometric analyses of variables measured longitudinally, such as sensitivity to change and responsiveness, were presented. It is also important to consider not only the Spanish spoken in Spain, so it would be important for future studies to include Hispanic/Latino speaking participants to resolve any cultural differences with Spanish participants. Finally, the questionnaires were always provided in the same order, which may be a potential source of bias.

## Conclusions

The AAOS-FAMSp demonstrated high internal consistency, reliability and criterion validity. It is an instrument that can be introduced into the Spanish-speaking environment to be used by clinicians and researchers as a tool to assess and monitor their patients.

The AAOS-FAM questionnaire was translated and cross-culturally adapted to Spanish. The psychometric properties of the AAOS-FAMsp were reported, indicating satisfactory and consistent results with the original version. However, the factor structure was slightly different than the original AAOS-FAM questionnaire.

## Abbreviations

AAOS-FAM, American Academy of Orthopaedic Surgeons-Foot and Ankle Module; AAOS-FAMsp, American Academy of Orthopaedic Surgeons-Foot and Ankle Module (Spanish version); EML, extraction by maximum likelihood; FFI, Foot Function Index; FHSQ, Foot Health Status Questionnaire; ICC, interclass correlation; MDC90, minimal detectable change 90; PROM, patient reported outcome measures; SD, standard deviation; SEM, standard error of measurement

## Additional files

Additional file 1: The AAOS-FAM was translated into Spanish and culturally adapted to provide the new AAOS-FAMsp. (PDF 123 kb) Additional file 2: Raw data base containing descriptive results variables. (SAV 14 kb)
