# Supplementary material for: Cross-cultural adaptation and validation of the Spanish version of the American Academy of Orthopaedic Surgeons-Foot and Ankle Module (AAOS-FAMsp)
Source: J Orthop Surg Res. 2016 Jul 6;11:74. doi: 10.1186/s13018-016-0409-7 (PMC4936313; doi:10.1186/s13018-016-0409-7)
Supplement: Additional file 1: — The AAOS-FAM was translated into Spanish and culturally adapted to provide the new AAOS-FAMsp. (PDF 123 kb) [file 13018_2016_409_MOESM1_ESM.pdf]

Foot and Ankle

Versión Española

Pie y Tobillo

Cuestionario de resultados

Basado en la Version 2.0 Foot and Ankle Outcomes Instrument Revised, renumbered, reformatted August 2005

Fecha de hoy: / /

¡Gracias por completar este cuestionario! Nos ayudará a entender mejor tu salud general y cualquier problema relacionado con enfermedades óseas y musculares.

El cuestionario es absolutamente voluntario y tus respuestas serán estrictamente confidenciales.

Por favor, responde a todas las preguntas. Algunas preguntas pueden parecerse a otras, pero cada una es distinta. No hay respuestas correctas o incorrectas. Si no tienes claro cómo responder a una pregunta, intenta dar la mejor respuesta que puedas. Puedes hacer comentarios en el margen de la página. Leeremos todos los comentarios que hagas, así que puedes anotar todos los que quieras.

Fecha de Nacimiento: / /

Foot and Ankle

Versión Española

INSTRUCCIONES

Por favor responde a las siguientes preguntas, sobre el pie/tobillo que está siendo tratado o en seguimiento. Si se trata de AMBOS pies/tobillos, por favor responde a las preguntas pensando en el lado que esté **peor**. Todas las preguntas se refieren a cómo te has encontrado, en general, durante la **semana pasada**.
Si estás recibiendo tratamiento por una lesión que ocurrió hace menos de una semana, por favor responde a las preguntas teniendo en cuenta el tiempo que ha pasado desde la lesión.

	Nada	Ligeramente	Moderadamente	Mucho	Extremadamente
1. **Durante la última semana, ¿cómo de rígido ha estado tu pie/tobillo?** (Rodea una de las siguientes respuestas.)	1	2	3	4	5
2. **Durante la última semana, ¿cómo de hinchado ha estado tu pie/tobillo?** (Rodea una de las siguientes respuestas.)	1	2	3	4	5

Durante la **última semana**, por favor dinos cuánto te ha dolido el pie/tobillo durante las siguientes actividades:
(Rodea UNA respuesta en cada línea, respondiendo a tu habilidad general para hacer estas actividades).

	Nada doloroso	Ligeramente doloroso	Moderadamente doloroso	Muy doloroso	Extremadamente doloroso	No pude hacer esta actividad a causa del dolor en el pie/tobillo	No pude hacer esta actividad por otros motivos
3. **¿Caminar sobre superficies desniveladas?**	1	2	3	4	5	6	7
4. **¿Caminar sobre superficies planas?**	1	2	3	4	5	6	7
5. **¿Estando tumbado/a en la cama de noche?**	1	2	3	4	5	6	7
6. **¿Subir o bajar escaleras?**	1	2	3	4	5	6	7

Foot and Ankle

Versión Española

Durante la **última semana**, se dobló tu pie/tobillo durante las siguientes actividades. (Rodea UNA respuesta en cada línea, la que mejor te describa para cada nivel de actividad).

		No se dobló en absoluto	Se dobló parcialmente, pero no me caí.	Se dobló completamente, así que me caí.	No pude hacerlo porque se me dobló el pie/tobillo.	No pude hacerlo por otros motivos.
**7.**	**Actividad fuerte;** ¿trabajo físico pesado, esquiar, tenis?	**1**	**2**	**3**	**4**	**5**
**8.**	**Actividad moderada;** ¿trabajo físico moderado, footing, correr**?**	**1**	**2**	**3**	**4**	**5**
**9.**	**Actividad leve,** ¿caminar, tareas de la casa, trabajo de jardinería?	**1**	**2**	**3**	**4**	**5**

10. ¿Cuál de las siguientes afirmaciones es la que mejor describe tu capacidad para moverte la mayor parte del tiempo en la última semana? (Rodea una respuesta).

1. No necesité nada de apoyo o ayuda.
2. Pude andar la mayor parte del tiempo sin apoyo o ayuda.
3. Usé un bastón o una muleta la mayor parte del tiempo para ayudarme a desplazarme.
4. Usé dos bastones, dos muletas o un andador la mayor parte del tiempo para ayudarme a desplazarme.
5. Usé una silla de ruedas.
6. Usé otros apoyos o alguien tuvo que ayudarme a desplazarme la mayor parte del tiempo.
7. No fui capaz de desplazarme en absoluto.

11. ¿Cuánta dificultad tuviste para mantener el equilibrio en la última semana? (Rodea una respuesta).

1. Nada de dificultad
2. Un poco de dificultad
3. Dificultad moderada
4. Bastante dificultad
5. Mucha dificultad
6. No puedo mantener el equilibrio para nada

12. **¿Cuánta dificultad tuviste en la última semana para ponerte o quitarte los calcetines o las medias?** (Rodea una respuesta).

1. Nada de dificultad
2. Un poco de dificultad
3. Dificultad moderada
4. Bastante dificultad
5. Mucha dificultad
6. No puedo hacerlo

Todas las preguntas son sobre cómo te has encontrado en general **durante la última semana**. Durante la última semana, por favor coméntanos cuánto dolor sentiste cuando estuviste realizando las siguientes actividades. (Rodea UNA de las siguientes respuestas en cada línea para describir lo mejor posible tu capacidad en general.)

	Nada de dolor	Dolor Leve	Dolor Moderado	Dolor Severo	Dolor extremo	No pude hacerlo a causa del dolor de pie/tobillo	No pude hacerlo por otros motivos
**13. Actividad fuerte; ¿trabajo físico pesado, esquiar, tenis?**	1	2	3	4	5	6	7
**14. Actividad moderada; ¿trabajo físico moderado, footing, correr?**	1	2	3	4	5	6	7
**15. Actividad leve, ¿caminar, tareas de la casa, trabajo de jardinería?**	1	2	3	4	5	6	7
**16. Permanecer de pie durante una hora**	1	2	3	4	5	6	7
**17. Permanecer de pie durante unos minutos**	1	2	3	4	5	6	7

18. **¿Cuánta dificultad tienes para caminar sobre superficies desniveladas** (por ejemplo: gravilla, piedras o un terreno inclinado)? (Rodea una respuesta).

1. Nada de dificultad
2. Un poco de dificultad
3. Dificultad moderada
4. Dificultad severa
5. Dificultad extrema
6. No puedo hacerlo por el pie/tobillo
7. No puedo hacerlo por otros motivos

¿Qué tipo de zapatos puedes usar cómodamente? (Rodea una respuesta en cada línea.)

		SI	NO	No se puede aplicar en mi caso
19.	**Cualquier zapato de mujer (incluyendo tacones altos) O cualquier zapato de hombre (incluyendo zapatos de vestir).**	1	2	3
20.	**La mayoría de los zapatos de vestir de mujer (excepto tacones altos) O la mayoría de los zapatos de hombre.**	1	2	3
21.	**Zapatillas deportivas, de paseo o calzado informal.**	1	2	3
22.	**Zapatos ortopédicos o de prescripción médica**	1	2	3
23.	**Todos los zapatos**	1	2	3

24. **¿Cuánto ha interferido tu problema de pie o tobillo con tu trabajo habitual, incluyendo tanto el trabajo fuera de casa como las tareas del hogar?** (Rodea una respuesta).

1. Nada
2. Un poco
3. Algo
4. Bastante
5. Muchísimo
6. Fui incapaz de trabajar debido a los problemas del pie/tobillo

25. **¿Cuánto interfiere tu problema de pie o tobillo con tu vida y en la capacidad que tienes para hacer lo que quieras?** (Rodea una respuesta).

1. Nada
2. Un poco
3. Algo
4. Bastante
5. Muchísimo
6. Lo estropea todo

MUCHAS GRACIAS POR COMPLETAR ESTE CUESTIONARIO
